# Small Leucine-Rich Proteoglycan *PODNL1* Identified as a Potential Tumor Matrix-Mediated Biomarker for Prognosis and Immunotherapy in a Pan-Cancer Setting

**DOI:** 10.3390/cimb45070386

**Published:** 2023-07-22

**Authors:** Geyang Dai, Yue Sun, Rui Wei, Ling Xi

**Affiliations:** 1 Cancer Biology Research Center, Key Laboratory of the Ministry of Education, Tongji Hospital, Tongji Medical College, Huazhong University of Science and Technology, Wuhan 430030, China; daigeyang1995@163.com (G.D.); 17803853986@163.com (Y.S.); weirui2018@tjh.tjmu.edu.cn (R.W.); 2 Department of Gynecologic Oncology, Tongji Hospital, Tongji Medical College, Huazhong University of Science and Technology, Wuhan 430030, China

**Keywords:** podocan-like protein 1 (PODNL1), pan-cancer, small leucine-rich proteoglycans (SLRPs), extracellular matrix (ECM), matrix-mediated biomarker, immunotherapy

## Abstract

The podocan-like protein 1 (PODNL1), an important member of the small leucine-rich proteoglycans (SLRP) family, is a crucial component of the tumor microenvironment (TME). But its prognostic values and the role in the TME have not been systematically estimated in a pan-cancer setting. Targeting *PODNL1*, a systematic exploration into the TCGA datasets, reconciling with the analyses of single-cell transcriptomes and immunotherapeutic cohorts in cancers, and validation by tissue microarray-based multiplex immunofluorescence staining was performed. PODNL1 was significantly correlated with the poor prognosis and immunotherapeutic responses in various cancers. In-depth demonstration of molecular mechanisms indicated that *PODNL1* expressions were notably positively correlated with cancer-associated fibroblast (CAF) infiltration levels in 33 types of cancers. It also positively correlated with the pan-fibroblast TGF-β response signature score, and the hallmarks including TGF-β, TNF-α, inflammatory response, apical junction, epithelial–mesenchymal transition and hedgehog in pan-cancer. Furthermore, high *PODNL1* expressions were positively related with the regulation of tumor-promoting TGF-β signaling through downregulating SMAD2/3:4 heterotrimer regulations transcription and up-regulating the pathway restricted SMAD protein phosphorylation. Single-cell transcriptome analyses and immunofluorescence validations indicated that *PODNL1* was predominantly expressed in the cancer cells and CAFs in various cancers. Additionally, the heterogeneity of cancer genotype–phenotype cross-talking was also observed associated with *PODNL1*. Our systematic study indicates that *PODNL1* plays an important role in the complex regulation network of tumor progression, and lays a foundation for further exploration to develop *PODNL1* as a valuable matrix-mediated biomarker for cancer immunotherapy and prognosis in a pan-cancer setting.

## 1. Introduction

The major complexity and heterogeneity of cancer genotype–phenotype cross-talking are inviting an elevated exploration of cancer hallmarks and are giving rise to the integrative concept for establishing a knowledge system of cancer [1]. Although prominently utilized for cancer treatment, cancer immunotherapy, especially of the immune checkpoint blockade [2], when being contextualized within the tumor microenvironment (TME), such as when combined with the matrix-mediated therapy, is paving a new avenue for conquering cancer in a pan-cancer setting. In recent years, the extracellular matrix (ECM) has been increasingly recognized to play a crucial role in multiple processes in tumor progression, metastasis and especially immune evasion. Some ECM proteins can not only change the TME at the topographical, architectural, biomechanical, and biochemical levels, but also serve as “matricryptins” which contain cryptic domains acting functions like chemokines and cytokines, regulating and transducing crucial signaling pathways within the TME. During carcinogenesis, such ECM proteins become highly dysregulated, emerging as a reservoir of the cancer markers associated with prediction, diagnosis, and prognosis, and as a pool of novel targets for matrix-mediated therapy as well [3].

Small leucine-rich proteoglycans (SLRPs) comprising five classes (I–V) with leucine-rich repeat (LRR) motifs are involved in the ECM assembly and related with various biological processes including the modulation of collagen fibrillogenesis and tumor progression [4,5]. In some breast cancer models, downregulated SLRPs of classes I and II, e.g., decorin and lumican, are demonstrated to be correlated with tumor-suppressive function [6,7], and in other models, high levels of lumican in non-metastatic pancreatic cancer are found to be associated with a more quiescent cancer cell state and prolonged patient survival [8]. In another trial with colorectal adenocarcinoma, lumican is shown to have close correlations with the infiltration levels of immune cells including the tumor-associated macrophages, regulatory T cells, and dendritic cells [9]. Meanwhile, another class I member biglycan secreted by the cancer-associated fibroblasts (CAFs) is demonstrated to cause a poor prognosis and be associated with the immunosuppressive TME [10], and high biglycan expression is also found to be associated with tumor invasiveness in several types of cancer [11]. Also, previous studies show that the main SLRP members, including decorin, biglycan, asporin, and fibromodulin, are able to bind to and regulate the transforming growth factor beta (TGF-β) pathway [4] and, among them, decorin modulates TGF-β signaling via interacting with the low-density lipoprotein receptor-related protein (LRP-1) and affects the mechanical dynamics of three-dimensional collagen matrices [12,13].

**Figure 1:**
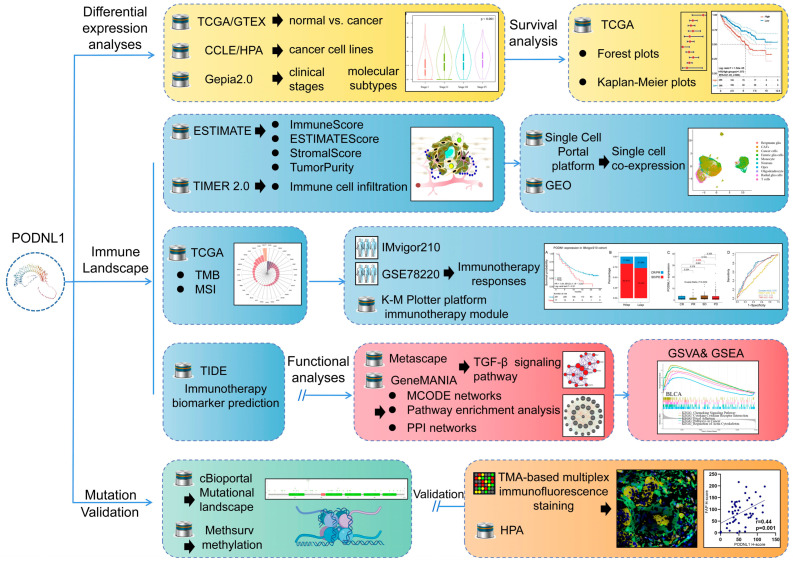
The workflow and experimental framework of this study. Targeting PODNL1, a systematic exploration into the datasets including TCGA, reconciling with the analyses of single-cell transcriptomes and immunotherapeutic cohorts in cancers was performed, aiming to evaluate the correlations between PODNL1 differential expression levels and prognostic value in a pan-cancer setting, and further systematically explore the related immune mechanism contextualizing the tumor environment and immunotherapy responses. Further validation was conducted by tissue microarray-based multiplex immunofluorescence staining. All the abbreviations are listed in Table S1.

In particular, SLRPs have a class V member, named podocan-like 1 (PODNL1), which is found to be highly expressed in bone, glioma, bladder and ovarian cancers, representing a potential prognostic biomarker [14,15,16,17,18]. Its high expression together with the presence of low-methylation in its CpG sites is coupled with significantly increased expressions of *PD-1*, *PD-L1*, and *CTLA4* in glioma [17]. Its overexpression in glioma cells is even believed to remarkably promote the mesenchymal-related biomarkers including vimentin, N-cadherin, snail, and fibronectin [16]. However, its prognostic values and the role in the TME have not been systematically estimated yet, especially in a pan-cancer setting. Therefore, in the present study, we performed a systematic exploration of *PODNL1* through the Cancer Genome Atlas (TCGA) pan-cancer datasets, TIMER (Tumor Immune Estimation Resource), Gene Set Variation Analysis (GSVA), Gene Set Enrichment Analysis (GSEA), and the Cellminer, reconciling with the analyses of single-cell transcriptomes and immunotherapeutic cohorts in cancers, and validation by the tissue microarray (TMA)-based multiplex immunofluorescence staining in 20 types of cancer as well (Figure 1).

## 2. Materials and Methods

### 2.1. Data Collection and Differential Expression Analyses

The clinical stages and survival information including overall survival time in days (OS), disease-free survival (DFS), disease-specific survival (DSS), progression-free survival (PFS), RNA-seq and SNP data of 33 types of cancers were acquired from the TCGA database (https://portal.gdc.cancer.gov/ (accessed on 5 April 2022)). Meanwhile, the information of normal samples was collected from the Genotype–Tissue Expression (GETx) dataset, conflated with TCGA data and then corrected to calculate the pan-cancer differences of PODNL1 expressions. The cell line expression matrix was downloaded from the Cancer Cell Line Encyclopedia (CCLE) database (https://portals.broadinstitute.org/ccle/ (accessed on 5 April 2022)) and Human Protein Atlas (HPA) database (https://www.proteinatlas.org/ (accessed on 6 April 2022)). The single-cell sequencing datasets of glioblastoma multiforme (GBM) were collected from the Single Cell Portal platform (http://singlecell.broadinstitute.org (accessed on 17 May 2022)) and other related single-cell sequencing datasets were collected from the Gene Expression Omnibus (GEO) database (https://www.ncbi.nlm.nih.gov/geo/ (accessed on 17 May 2022)) including bladder urothelial cancer (BLCA, GSE145137), head and neck squamous cell carcinoma (HNSC, GSE103322), kidney renal clear cell carcinoma (KIRC, GSE121636 and GSE171306), ovarian serous cystadenocarcinoma (OV, GSE118828). The analyses of the PODNL1 differential expressions within tumor subtypes were conducted by The Gene Expression Profiling Interactive Analysis (GEPIA2, http://gepia.cancer-pku.cn (accessed on 10 April 2022)) subtype filter. All the abbreviations in this paper are listed in Table S1.

### 2.2. Correlations between PODNL1 Expressions and Prognosis

The correlations between the PODNL1 expression levels and OS, DFS, DSS, and PFS in patients with cancers were evaluated using the Cox proportional hazards model and Kaplan–Meier (K-M) survival method through the R packages “survival”, “survminer”, and “forestplot”. The “timeROC” (v 0.4) R package was used to generate the receiver operating characteristic (ROC) curve to compare the predictive accuracy of PODNL1 expressions.

### 2.3. Tumor Immune Infiltration

The correlations between immune infiltration scores (including Immune Score, stromal score, estimate score, tumor purity) and the expression levels of PODNL1 were evaluated using ESTIMATE (estimation of stromal and immune cells in malignant tumor tissues using expression data) [19] in pan-cancer. The TIMER database (http://timer.cistrome.org/ (accessed on 2 June 2022)), which provides multiple immune deconvolution methods including TIMER, cell-type identification by estimating relative subsets of RNA transcripts (CIBERSORT), CIBERSORT-ABS, xCELL, estimating the proportion of immune and cancer cells (EPIC), the microenvironment cell populations counter (MCP-counter), a method to quantify the fractions of ten immune cell types from bulk RNA-sequencing data (quanTIseq), and TIDE algorithms were applied for analyzing immune infiltration levels [20].

### 2.4. The TME-Related Biological Processes

To explore the correlations between the expression levels of PODNL1 and TME, we collected the related gene signatures including the pan-fibroblast TGF-β response signature scores (Pan_F_TBRs), antigen processing machinery (APM), DNA repair, angiogenesis, and homologous recombination [21] as well as signatures of tumor inflammation, tumor proliferation, G2M checkpoint, epithelial–mesenchymal transition (EMT), and DNA replication [22]. In the following, we performed the PODNL1 correlation analyses with immunomodulators including inmmunostimulator, immunocheckpoint, major histocompatibility complex (MHC), chemokine and its receptors using the gene sets from an integrated repository portal for tumor–immune system interactions (TISDB, http://cis.hku.hk/TISIDB/ (accessed on 10 June 2022)), and with *TGF*-β gene sets from combined hallmark_TGF_beta_signaling and KEGG_TGF_beta_signaling_pathway genes from the molecular signatures database (MsigDB) [23]. The cancer stem cell markers gene set was summarized from the previous study [24]. The multi-gene correlation was analyzed by the R software package “pheatmap”. Cancer hallmark signatures were analyzed by the GSVA method and Kyoto Encyclopedia of Genes and Genomes database (KEGG) terms by the GSEA. Finally, the protein–protein interaction (PPI) network was analyzed using GeneMANIA (http://www.genemania.org (accessed on 13 June 2022)).

### 2.5. Tumor Mutation Burden (TMB) and Microsatellite Instability (MSI)

Though defined as the total amount of somatic genetic-coding errors, base substitutions, insertions or deletions detected per mega base, TMB in this study was determined by calculating the variant frequency and the number of variants/exon length for each tumor sample after dividing the non-synonymous mutation sites by the total length of the protein-coding region. The value of MSI for each TCGA patient was derived from previously published studies [25]. The correlations of the expression levels of PODNL1 with TMB and MSI were analyzed using Spearman’s rank correlation coefficient, and then visualized by the “Fmsb”, “Limma” and “Dplyr” R packages.

### 2.6. Mutation Landscape and Methylation

The pan-cancer mutation landscape of *PODNL1* was measured using cBioportal (www.cbioportal.org (accessed on 4 July 2022)). The Methsurv database (https://biit.cs.ut.ee/methsurv (accessed on 6 July 2022)) was used to find the methylation sites and prognostic information of *PODNL1* in pan-cancer. The correlations of *PODNL1* methylation with the cytotoxic T lymphocyte (CTL) and T cell dysfunction were analyzed via the Tumor Immune Dysfunction and Exclusion platform (TIDE) [26].

### 2.7. The Immunotherapeutic Responses

The IMvigor210 cohort [21] of patients with metastatic urothelial cancer (mUC) receiving anti-PD-L1 immunotherapy (http://research-pub.gene.com/IMvigor210CoreBiologies (accessed on 9 July 2022)), and the GSE78220 cohort [27] of patients with melanoma receiving anti-PD-1 immunotherapy were used to further analyze the expression levels of PODNL1 with the immunotherapeutic responses. The immunotherapeutic responses, respectively, were represented as the complete response (CR), partial response (PR), stable disease (SD), and progressive disease (PD). The prognosis Risk, Risk.adj and ROC for *PODNL1* expressions in immunotherapeutic cohorts were calculated by the TIDE platform [26]. The OS K-M curves for the cohorts receiving anti-CTLA4 and anti-PD-1 treatments were analyzed by K-M Plotter platform immunotherapy module (https://kmplot.com/analysis/index.php?p=service&cancer=immunotherapy (accessed on 11 July 2022)).

### 2.8. Analyses of Single-Cell Transcriptomes and Drug Responses

The R package “Seuratv4.1.1.” was applied for basic analyses of single-cell RNA sequencing data, including data filtering, quality control and integrating. The corrected normalized data metrics were applied to the standard analysis. Principal component analysis (PCA) was carried out for dimension reduction. Then, cell clustering was performed using the FindClusters function implemented in the “Seurat” R package. The R package “Infercnv” and “Copykat” were used for the identification of tumor cells. Dimensionality reduction was visualized using the uniform manifold approximation and projection (UMAP) function and *PODNL1* gene expression levels were plotted by “Vlnplot”, “Dimplot” and “Featureplot”. The correlations of the expression levels of PODNL1 with drug responses in pan-cancer treatment were analyzed using the Cellminer datasets (http://discover.nci.nih.gov/cellminer/ (accessed on 15 July 2022)).

### 2.9. Multiplex Immunofluorescence Staining

The digital image analyses (DIA) of TMA-based multiplex immunofluorescence staining were performed as described by previous studies [28]. TMA chips containing a total of 52 pairs of tumor and matched adjacent normal tissues from 20 types of cancer including LUAD, LUSC, small-cell lung cancer (SCLC), BLCA, KIRC, BRCA, THCA, GBM, PRAD, ESCA, STAD, COAD, READ, PAAD, LIHC, CESC, UCEC, OV, SKCM, and DLBC were obtained from Shanghai Zhuoli Biotechnology Company Ltd., Shanghai, China. Two pathologists reviewed the hematoxylin-andeosin (HE)-stained tumor, whole sections of formalin-fixed, paraffinembedded (FFPE) specimens and identified regions of invasive carcinoma, which displayed the typical histological features for sampling into TMAs. For each sampling pair, one to three representative tumor cores and one core with normal or tumor-adjacent tissue with a 1.5 mm diameter were selected. The primary monoclonal antibodies were against PODNL1 (206269-T08, 1:50, SinoBiological, Shanghai, China) and FAP (BM5121, 1:100, Boster, Wuhan, China). Then, the DIA technology using the fully automated VIS DIA VisioMorph system (Visiopharm^®^, Hoersholm, Denmark) was applied to evaluate the relationship between PODNL1 and fibroblast activation protein α (FAP) expression levels, while the positive cells were quantified at a single-cell level. The histochemistry score is given by (H-Score) =∑ (*pi* × *i*) = (percentage of weak intensity × 1) + (percentage of moderate intensity × 2) + (percentage of strong intensity × 3), *i* stands the staining intensity and *pi* represents the ratio of positive cells in the section.

### 2.10. Statistical Analysis

All analysis methods and R packages were implemented by R version 4.0.3., with a *p* value < 0.05 considered as statistically significant. Correlations of two variables were analyzed using Spearman’s or Pearson’s test. In the case of two groups’ comparisons, Student’s *t*-test and the Wilcoxon rank-sum test were used for normally and nonnormally distributed variables, respectively. For the comparisons of more than two groups, the Kruskal–Wallis tests and one-way analysis of variance were, respectively, utilized as the nonparametric and parametric methods.

## 3. Results

### 3.1. mRNA Expression and Prognostic Value

**Figure 2:**
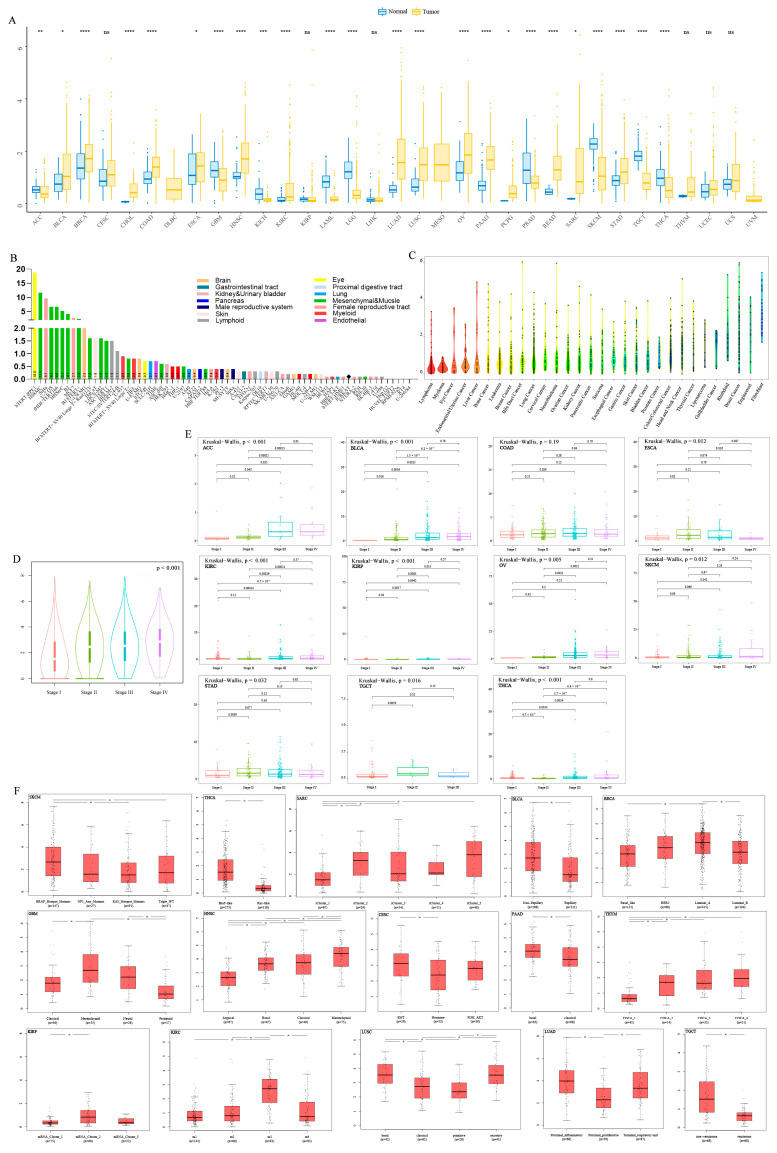
Differential expression analyses of *PODNL1* among cancers. (**A**) Expression profiles of *PODNL1* analyzed with TCGA and GTEX databases. (**B**) The expression levels of PODNL1 in tumor cell lines in CCLE database. (**C**) The expression levels of PODNL1 in 69 cell lines from the HPA database. (**D**) The correlations between the *PODNL1* expressions and the clinical stages in 33 types of cancers. (**E**) The correlations between the *PODNL1* expressions and the clinical stages in ACC, KIRC, KIRP, THCA, KICH, OV, BLCA, SKCM, COAD, STAD, ESCA, and TGCT. (**F**) The respective analyses of correlations between the *PODNL1* expressions and the molecular subtypes in SKCM, THCA, SARC, BLCA, TGCT, GBM, CESC, HNSC, PAAD, LUAD, KIRP, KIRC, LUSC, THYM, and BRCA. * *p* < 0.05, ** *p* < 0.01, *** *p* < 0.001, **** *p* < 0.0001. All the abbreviations are listed in Table S1.

The expression levels of PODNL1 were found significantly higher in tumor tissues in 15 types of cancers including BLCA, breast-invasive carcinoma (BRCA), cholangiocarcinoma (CHOL), colon adenocarcinoma (COAD), esophageal carcinoma (ESCA), HNSC, KIRC, lung adenocarcinoma (LUAD), lung squamous cell carcinoma (LUSC), OV, pancreatic adenocarcinoma (PAAD), pheochromocytoma and paraganglioma (PCPG), rectum adenocarcinoma (READ), sarcoma (SARC), and stomach adenocarcinoma (STAD), compared with in normal tissue samples. Meanwhile, the expression levels of PODNL1 in nine types of cancers were significantly downregulated (Figure 2A). Further analyses indicated that *PODNL1* was expressed in different cancer cell lines in CCLE datasets, and higher in fibroblasts and brain tumor cells (Figure 2B), and its expression was enhanced in cell lines of fHDF/TERT166, HSkMC, hTERT-RPE1, SiHa and U-2-OS, which were derived from brain, muscle, female reproductive system, and mesenchymal tissues, respectively, in HPA datasets (Figure 2C). Analysis of clinical stages in pan-cancer presented the results that the expression levels of PODNL1 increased with tumor development as its expression levels differed significantly between early (I and II) and advanced (III and IV) stages in multiple cancers (Figure 2D,E). Moreover, analyses of molecular subtypes in GEPIA also indicated significant variation among the pan-cancer (Figure 2F). The high expression levels were correlated with the v-raf murine sarcoma viral oncogene homolog B1 (*BRAF*) mutation type of thyroid carcinoma (THCA) and skin cutaneous melanoma (SKCM), the mesenchymal/EMT phenotype of GBM, cervical squamous cell carcinoma and endocervical adenocarcinoma (CESC), HNSC, PAAD, LUSC and OV, the M3 type of KIRC, non-papillary type of BLCA, proximal-inflammatory phenotype of LUAD, luminal-A phenotype of BRCA, and a non-seminoma type of testicular germ cell tumors (TGCT).

**Figure 3:**
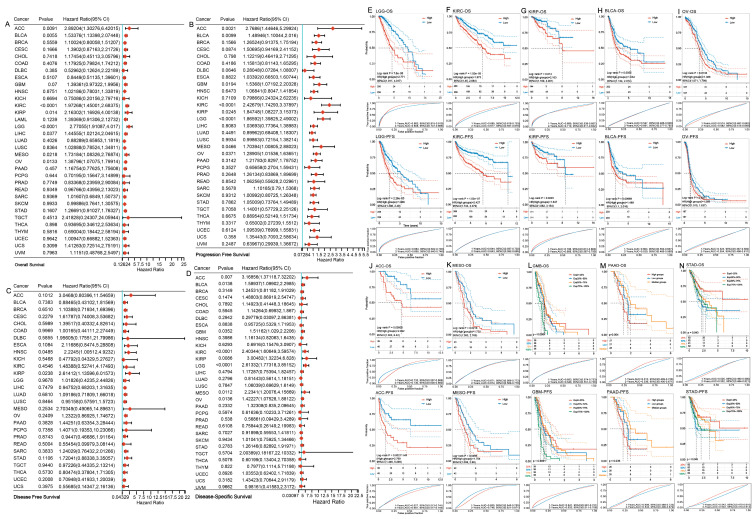
Pan-cancer survival analysis of *PODNL1* using TCGA database. Through analysis using univariate cox regression, the forest plots representing the *PODNL1* expressions significantly associated with the following: (**A**) Overall survival time in days (OS) in KIRC, LGG, BLCA, ACC, OV, LIHC, MESO and KIRP, which indicated that *PODNL1* was a high-risk gene in these cancers, particularly in ACC and KIRP. (**B**) Progression-free survival (PFS) in LGG, GBM, KIRC, ACC, BLCA, KIRP, OV, and MESO, which indicated that *PODNL1* was a high-risk gene, particularly in ACC and KIRC. (**C**) Disease-free survival (DFS) in HNSC and KIRP. (**D**) Disease-special survival (DSS) in ACC, LGG, KIRC, BLCA, GBM, KIRP, MESO, and OV. (**E**–**N**) The correlations of the high expression levels of PODNL1 with the poor OS and PFS of patients with cancers including LGG, KIRC, KIRP, BLCA, OV, ACC, MESO, GBM, PAAD metastasis and recurrence, and STAD Stage M0, analyzed by the Kaplan–Meier (**K**–**M**) method, and the ROC curves measuring their predictive diagnostic values of the *PODNL1* expressions.

Further exploration via the cox proportional hazards model demonstrated that significant correlations between the expression levels of PODNL1 and OS, PFS, DFS, and DSS in various cancers (Figure 3A–D). Poor OS, PFS, DFS, and DSS were all shown to be significantly correlated with higher expression levels in kidney renal papillary cell carcinoma (KIRP) (*p* < 0.05, HR > 1.8). Meanwhile, the K-M survival curves showed that, among the patients with 10 types of cancers including brain lower grade glioma (LGG), KIRC, KIRP, BLCA, OV, adrenocortical carcinoma (ACC), mesothelioma (MESO), GBM, PAAD with metastasis, and STAD in stage M0, the high PODNL1 expressions significantly related with both poor OS and PFS, with the correlated means of the area under the ROC curves (AUC) all larger than 0.5 (Figure 3E–N). Among them, LGG, ACC and KIRP had the AUC of both OS and PFI more than 0.7 (Figure 3E,G,J).

### 3.2. Immune Infiltration

**Figure 4:**
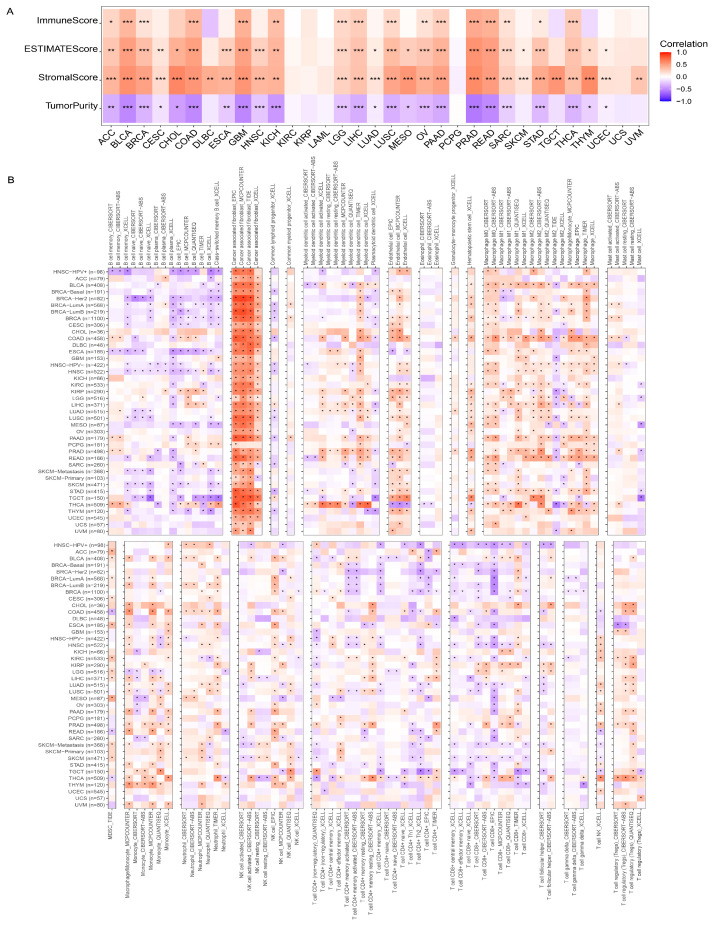
Correlation analyses between the *PODNL1* expressions and the infiltration levels of immune cells among pan-cancer. (**A**) Heatmap of the correlations between the expression levels of PODNL1 and Immune Score, ESTIMATEScore, StromalScore, and TumorPurity, respectively. (**B**) Heatmap of the correlations between the expression levels of PODNL1 and immune cells evaluated by the TIMER, CIBERSORT, CIBERSORT-ABS, XCELL, EPIC, MCPCOUNTER, QUANTISEQ, and TIDE algorithms. Red showed the positive correlations and purple the negative. * *p* < 0.05, ** *p* < 0.01, *** *p* < 0.001.

Significant correlations were observed between the expression levels of PODNL1 and immune infiltration at a pan-cancer scale (Figure 4). The expression levels of PODNL1 were positively correlated with Immune Score, ESTIMATE score and stromal score, and negatively with tumor purity in 16, 25, 28, and 24 types of cancers, respectively (Figure 4A). Further analyses presented significant correlations of the expression levels of PODNL1 with multiple TME components in pan-cancer, notably positively correlated with the infiltration levels of CAFs in all types of cancers, endothelial cells in 22 types of cancers, and hematopoietic stem cells (HSCs) in 19 types of cancers. In adaptive immune cells, the infiltration levels of CD8 + T cells were found negatively correlated with the *PODNL1* expressions in 13 types of cancers, and positively correlated in six types of cancers. The infiltration levels of CD4 + T cells were negatively correlated with PODNL1 expressions in HCSC and BRCA. The total CD4 + T cells were negatively correlated, but the Th2 cells positively correlated in BLCA. Moreover, the infiltration levels of B cells presented significant negative correlations with PODNL1 expressions in the majority of pan-cancer except THCA, KIRP, liver hepatocellular carcinoma (LIHC) and PCPG. In the innate immune cells, the infiltration levels of macrophages and monocytes were found positively correlated with the *PODNL1* expressions. In addition, *PODNL1* expressions showed significant positive correlation with myeloid-derived suppressor cells (MDSCs) in 11 types of cancers including ACC, BLCA, BRCA-luminal A, CESC, ESCA, KIRC, LGG, LIHC, MESO, SKCM, and SKCM metastasis (Figure 4B).

### 3.3. The Single-Cell Transcriptomes Analyses

**Figure 5:**
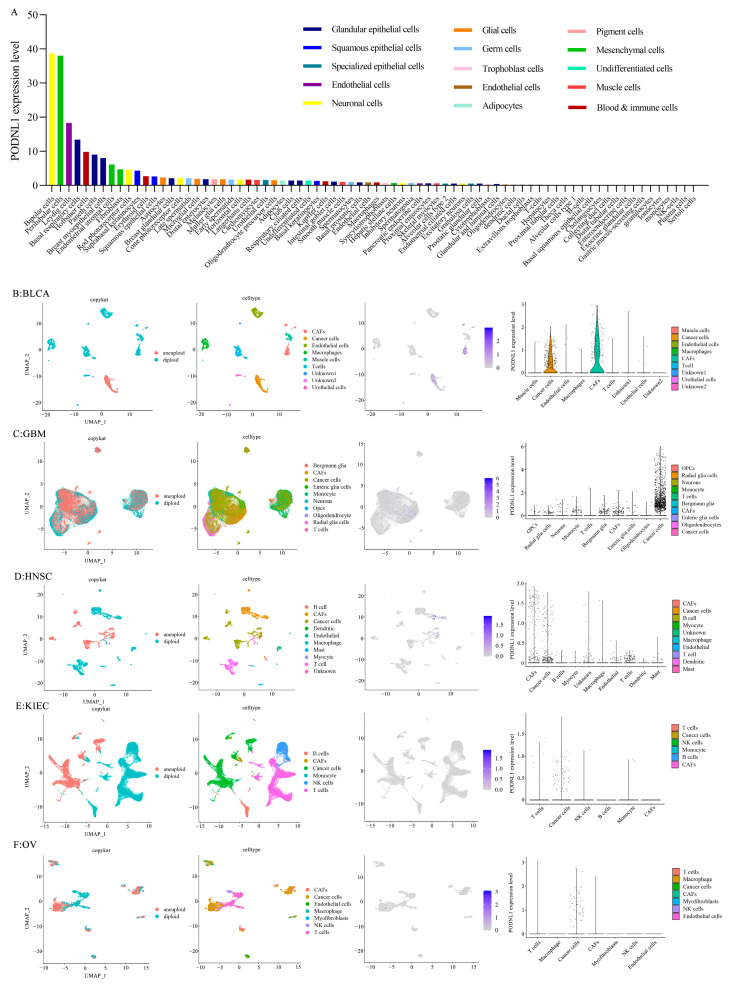
Single-cell sequencing analyzing *PODNL1* co-expression in pan-cancer. (**A**) The expression levels of PODNL1 in cells analyzed via the single-cell transcriptomes of normal tissues in HPA datasets. (**B**–**F**) The expression levels of PODNL1 analyzed via the single-cell sequencing datasets of BLCA, HNSC, GBM, KIRC, and OV, respectively.

The single-cell transcriptome analyses demonstrated that *PODNL1* was mainly expressed in neuronal cells and mesenchymal cells in normal tissues within the HPA database (Figure 5A). Further single-cell sequencing analyses in five cancers (BLCA, GBM, HNSC, KIRC and GBM) indicated that *PODNL1* was highly expressed in cancer cells in all included cancers as well as in CAFs of BLCA and HNSC. Moreover, moderate expressions were found in stromal cells including neuronal cells, monocytes and T cells in GBM as well as B cells, monocytes, macrophages, T cells and epithelial cells in HNSC (Figure 5B–F).

### 3.4. Cancer Immunotherapy Related Core Molecule Events

**Figure 6:**
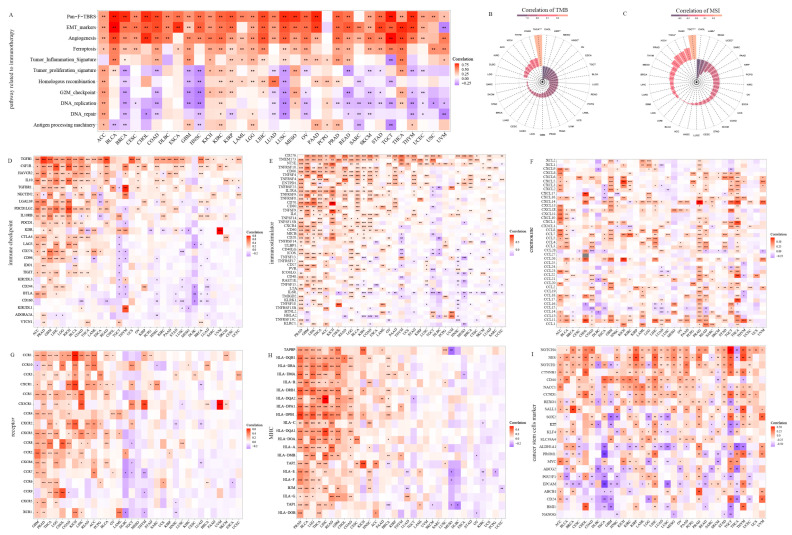
Correlations of *PODNL1* expressions with the core molecules and biology pathways related to tumor immunotherapeutic responses. Correlation heatmaps: (**A**) Pathways (including Pan_F_TBRs, EMT markers, angiogenesis, ferroptosis, tumor inflammation signature, tumor proliferation signature, homologous recombination, G2M checkpoint, DNA replication, DNA repair and antigen-processing machinery). (**B**) Immune checkpoint. (**C**) Immunostimulator. (**D**) Chemokine. (**E**) Chemokine receptor. (**F**) MHC. (**G**) Tumor stem cell marker. (**H**) TMB. (**I**) MSI. * *p* < 0.05, ** *p* < 0.01, *** *p* < 0.001.

A series of gene sets were adopted to examine the potential relevance of *PODNL1* expression to cancer immunotherapy. Among them, the Pan_F_TBRs, EMT and angiogenesis had significant positive correlations with *PODNL1* expressions in almost all the cancers (Figure 6A). Multiple types of cancers exhibited negative correlations with DNA replication (18), DNA repair (14) and G2-M_checkpoint (12) (Figure 6A). Further evaluation indicated that the highly expressed PODNL1 was significantly correlated with immune checkpoints, immunostimulators, chemokines and its receptors, MHC and stem markers (Figure 6B–G). Notably, immune checkpoints TGFB1 and CD276 were significantly positively correlated in 25 types of cancers (Figure 6B,C). Otherwise, cytokine receptors and MHC molecules were observed to heterogeneously aggregate in different tumors (Figure 6E,F). Also, *PODNL1* expressions were found to be positively correlated with the expression levels of cancer stem cell-related molecular markers as cluster of differentiation 44 (CD44), sex-determining region Y-box transcription factor 2 (SOX2), spalt-like transcription factor 1 (SALL1), RNA exonuclease 1 homolog (REXO1), nucleus accumbens-associated protein 1 (NACC1), cyclin D1 (CCND1), catenin beta-1 (CTNNB1), neurogenic locus notch homolog protein 1 (NOTCH1), NOTCH4, and nestin (NES) in more than 10 types of cancers, especially with the NES in more than 20 types of cancers (Figure 6G). Finally, in most tumors, *PODNL1* expressions were not significantly correlated with TMB and MSI, only both positively correlated in THCA, negatively with TMB in KIRP, HNSC, and negatively with MSI in TGCT and acute myeloid leukemia (LAML) (Figure 6H,I).

### 3.5. Correlations with Tumor-Promoting TGF-β Signaling

**Figure 7:**
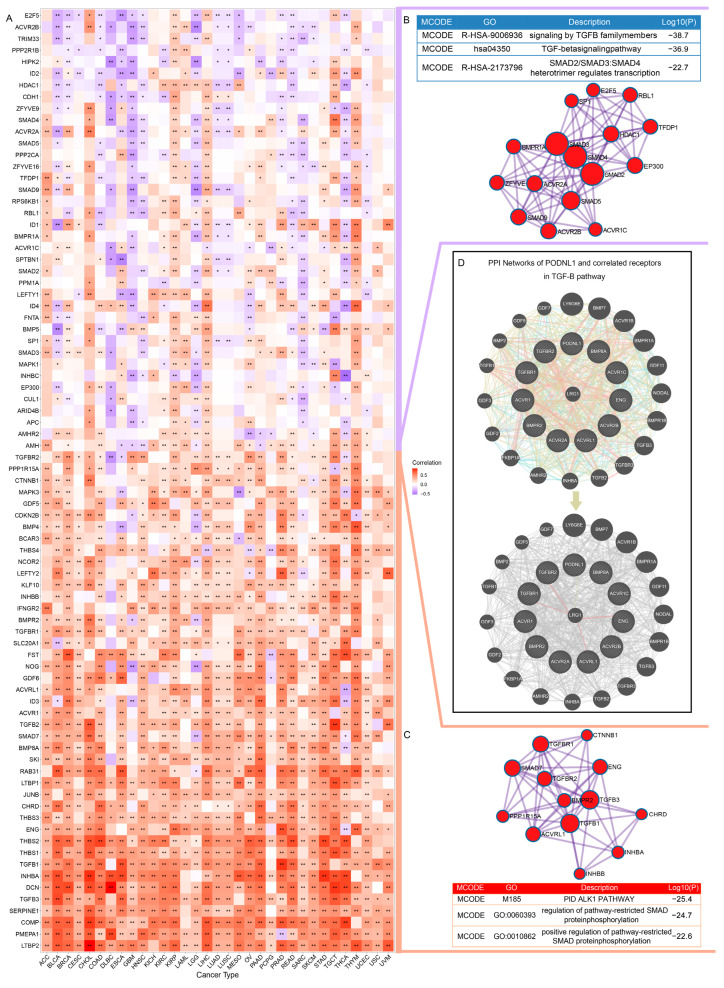
Correlations of *PODNL1* expressions with *TGF*-β signaling. (**A**) The correlations between *PODNL1* expressions and *TGF*-β signaling pathway genes. (**B**,**C**) The Metascape MCODE networks and the corresponding pathway enrichment analysis identified for positively related gene lists (in red column) and negatively ones (in purple column), respectively. (**D**) GeneMANIA protein–protein interaction (PPI) networks of PODNL1 were analyzed using its positively correlated receptors including TGFBR1, TGFBR2, ACVR1, ACVR1C, ACVR2A, ACVR2B, ACVRL1, BMP8A, BMPR2 and ENG. * *p* < 0.05, ** *p* < 0.01.

Because of the strong correlations of *PODNL1* expressions with CAFs infiltration levels and Pan_F_TBRs, further correlation analysis with *TGF*-β pathway genes was conducted and the results showed that multiple genes had significant correlations with its expressions (Figure 7A–C). On the whole, the majority of genes in the *TGF*-β pathway were positively correlated with *PODNL1* expression, while a few showed negative correlations (Figure 7A). We took the gene sets showing positive correlations in more than 20 types of tumors (red column in Figure 7A,C) and negative correlations in more than 5 ones (purple column in Figure 7A,B) to conduct the PPI analyses on the Metascape platform, respectively. In the molecular complex detection (MCODE) networks, the positively correlated genes were concentrated in the restricted SMAD protein phosphorylation pathway (Figure 7C) and the negatively correlated ones in the SMAD2/3:4 heterotrimer regulations pathway (Figure 7B). Since the proteoglycans SLRP family might play a role in regulating the *TGF*-β signaling through its receptors, further PPI analyses covering positively correlated receptors indicated that the PODNL1 protein shared a domain with leucine-richα-2 glycoprotein 1 (LRG1) which could physically interact with activin A receptor-like type 1 (ACVRL1), endoglin (ENG) and TGF beta receptor 1 (TGFBR1) (Figure 7D).

### 3.6. Immunotherapeutic Responses

**Figure 8:**
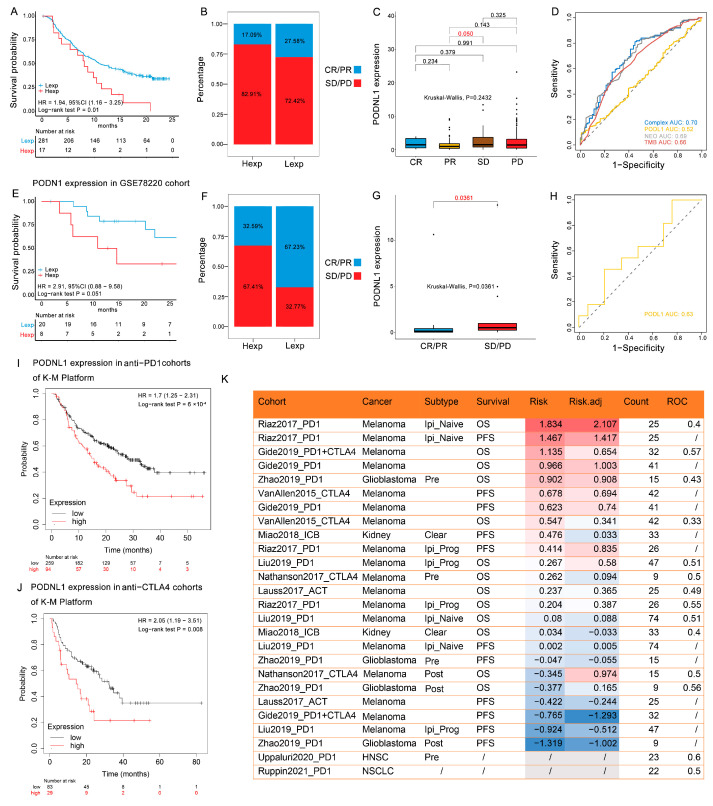
Immunotherapy responses and biomarkers related with the expression levels of PODNL1 in cancer immunotherapeutic cohorts. (**A**–**D**) The IMvigor210 cohort: (**A**) The K-M curve of OS. (**B**,**C**) Bar and box plots of immunotherapy responses (CR: complete response; PR: partial response; SD: stable disease; PD: progressive disease) and (**D**) the areas under the ROC curves (AUCs) of the PODNL1 expression, TMB, NEO, and their combination. (**E**–**H**) The GSE78220 cohort: (**E**) The K-M plot of OS. (**F**,**G**) Bar and box plots of immunotherapy responses (CR/PR/SD/ PD) and (**H**) the AUCs of the PODNL1 expressions. (**I**,**J**) The K-M curves of *PODNL1* expression in immunotherapy cohorts analyzed using the K-M plotter platform: (**I**) Anti-PD-1 cohorts. (**J**) Anti-CTLA4 cohorts. (**K**) The correlations of high *PODNL1* expressions with Risk, Risk.adj and ROC in immunotherapy cohorts enumerated in TIDE platform.

Further analyses suggested that the high expression levels of PODNL1 were significantly associated with the poor OS of patients from the enumerated immunotherapeutic cohorts (the IMvigor210 cohort of mUC treated with anti-PD-L1 (Figure 8A–D), the GSE78220 cohort with the melanoma treated with anti-PD-1 (Figure 8E–H), as well as the cohorts of the patients treated with anti-CTLA4 (Figure 8I), and the cohorts of the patients treated with anti-PD-1 (Figure 8J) immunotherapies, analyzed by the K-M platform. In addition, through the estimation of the biomarker relevance of *PODNL1* expressions within the cohorts of IMvigor210 and GSE78220, and 20 cohorts in the TIDE platform, we found that *PODNL1* expressions had the predictive diagnostic AUC of more than 0.5 in 11 cohorts among them (Figure 8K).

### 3.7. Correlations with Various Cancer Hallmarks via Functional Analyses

**Figure 9:**
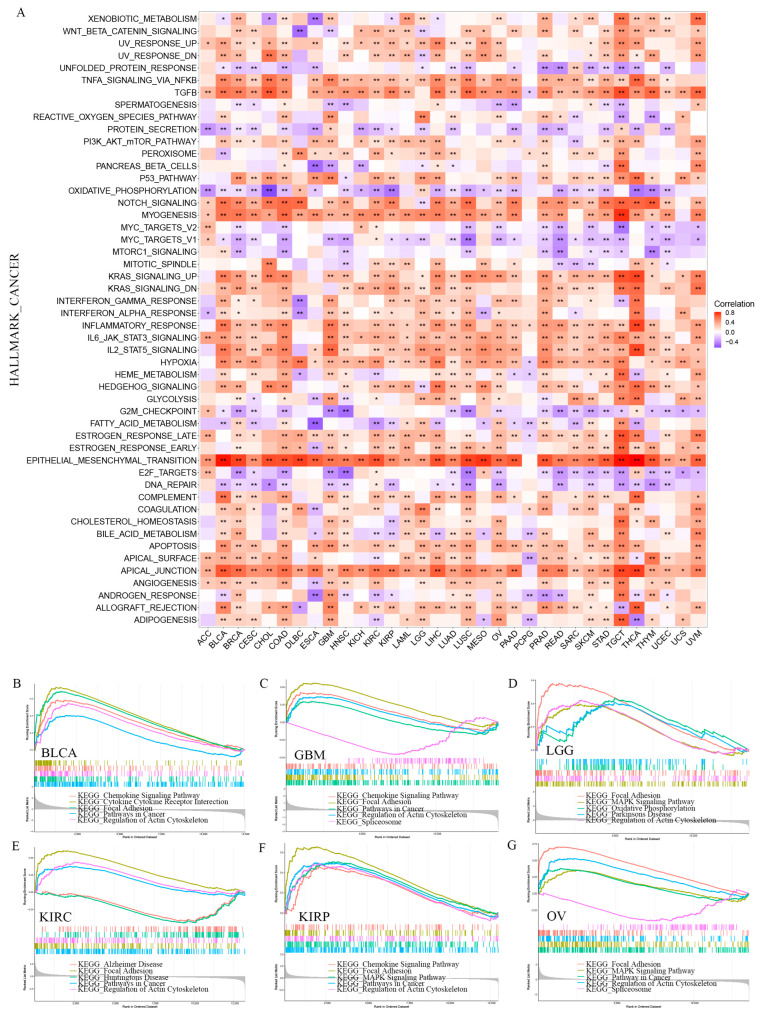
Functional analyses on *PODNL1* expressions among 33 types of cancers. (**A**) The correlated HALLMARK_CANCER in pan-cancer analyzed via GSVA. (**B**–**G**) Correlation analyses in BLCA, GBM, LGG, KIRC, KIRP, and OV via GSEA of KEGG. * *p* < 0.05, ** *p* < 0.01.

GSVA and GSEA were conducted to comprehensively investigate the related pathways with *PODNL1* expressions. GSVA on gene sets of the cancer hallmarks showed that the non-canonical *TGF*-β signaling pathway phosphoinositide 3 kinase-serine/threonine protein kinase–mechanistic target of rapamycin kinase (PI3K-AKT-mTOR), ECM-related pathways (including myogenesis, apical junction), the pathways associated with inflammation (including *TGF*-β, tumor necrosis factor-α (*TNF*-α), inflammatory response, interleukin 6-janus kinase-signal transductor of activators of transcription (IL-6-JAK-STAT3), IL-2-STAT5, and Hypoxia), and the pathways related to stemness (including EMT and hedgehog) had significant positive correlations with *PODNL1* expressions in various cancers. Notably, metabolism-related pathways, including fatty acid metabolism and adipogenesis, showed significantly negative correlations in multiple cancers (Figure 9A). The GSEA confirmed that *PONDL1* expressions significantly positively correlated with the pathways in cancers and focal adhesion in six types of cancers including LGG, GBM, KIRC, BLCA, KIRP and OV, which all exhibited a poor prognosis in the *PODNL1* high expression groups (Figure 9B–G).

### 3.8. Mutation and Methylation

**Figure 10:**
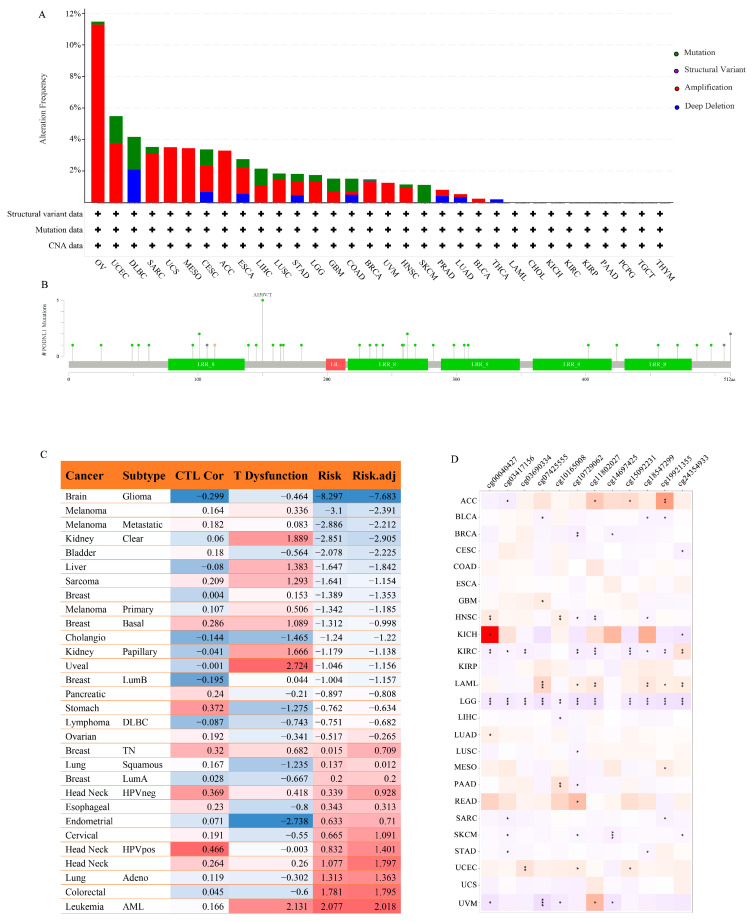
Mutational landscape and methylation of *PODNL1* in pan-cancer. (**A**) Mutation frequency of *PODNL1* in pan-cancer. (**B**) Mutation lollipop chart of *PODNL1*. (**C**) Correlation table of *PODNL1* methylation and CTL Cor, T Dysfunction, Risk and Risk.adj in 21 types of cancers from TCGA database, analyzed using the TIDE platform. (**D**) Correlation heatmap of *PODNL1* methylation sites and prognosis of 25 different human cancers from TCGA database, analyzed using the Methsurv platform. The red color represents a positive correlation while the purple negative. (* *p* < 0.05, ** *p* < 0.01, *** *p* < 0.001).

**Figure 11:**
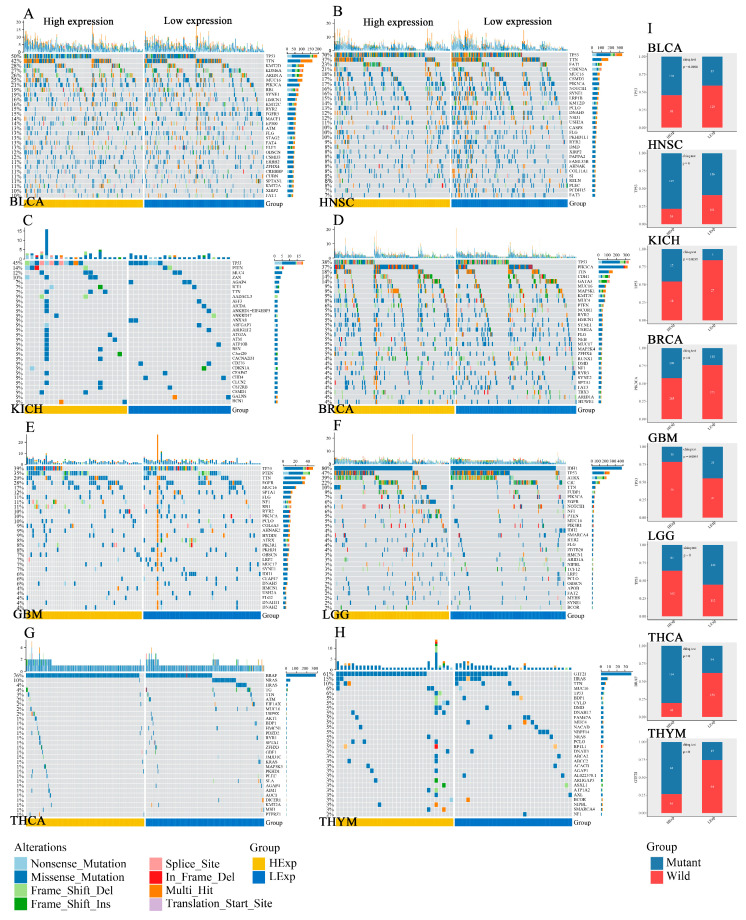
Oncoplots showed the somatic landscapes of (**A**) BLCA, (**B**) HNSC, (**C**) KICH, (**D**) BRCA, (**E**) GBM, (**F**) LGG, (**G**) THCA and (**H**) THYM tumor cohorts. Genes are ordered by their mutation frequencies, samples are ordered by disease histology, as indicated by the annotation bar. Waterfall plot shows mutation information for each gene for each sample. Color annotation of various cancer types are shown at the bottom. The bar plot above the legend shows the number of mutation burden. (**I**) The mutation profiles of different genes in high- and low-PODNL1-expression groups: *TP53* in BLCA, HNSC, KICH, GBM and LGG, *PIK3CA* in BRCA, *BRAF* in THCA and *GTF2I* in THYM.

The mutant landscape of *PODNL1* in pan-cancer showed that *PODNL1* altered mainly by amplification and mutation in OV, uterine corpus endometrial carcinoma (UCEC) and lymphoid neoplasm diffuse large B-cell lymphoma (DLBC) at a high level with a surprising alteration rate of more than 4%, especially in OV at the highest alteration rate of 11.47% (Figure 10A). A total of 36 mutation sites were found in PODNL1 protein, among which the site A150V/T had the highest incidence (Figure 10B). Furthermore, we found more *TP53* mutations in BLCA, HNSC, and KICH, and more mutations of phosphatidylinositol-4,5-bisphosphate 3-kinase catalytic subunit alpha (*PIK3CA*) in BRCA, more *BRAF* mutations in THCA, and more mutations of general transcription factor IIi (*GTF2I*) in thymoma (THYM) in PODNL1 high-expression groups. Contrarily, low *TP53* mutations were found to be associated with high PODNL1 expressions in GBM and LGG (Figure 11).

Analyses combining the TIDE platform and Methsurv Database demonstrated that the survival prognosis, CTL infiltration level and T cell dysfunction were affected by *PODNL1* methylation in multiple cancers, among which glioma was most significantly affected by methylation (Figure 10C,D). The low-level methylations in site Cg10729062 and site Cg03417156 were associated with poor prognosis in nine and six types of cancers, respectively (Figure 10D).

### 3.9. Analyses of the Genome-Wide Co-Expression and Drug Responses

**Figure 12:**
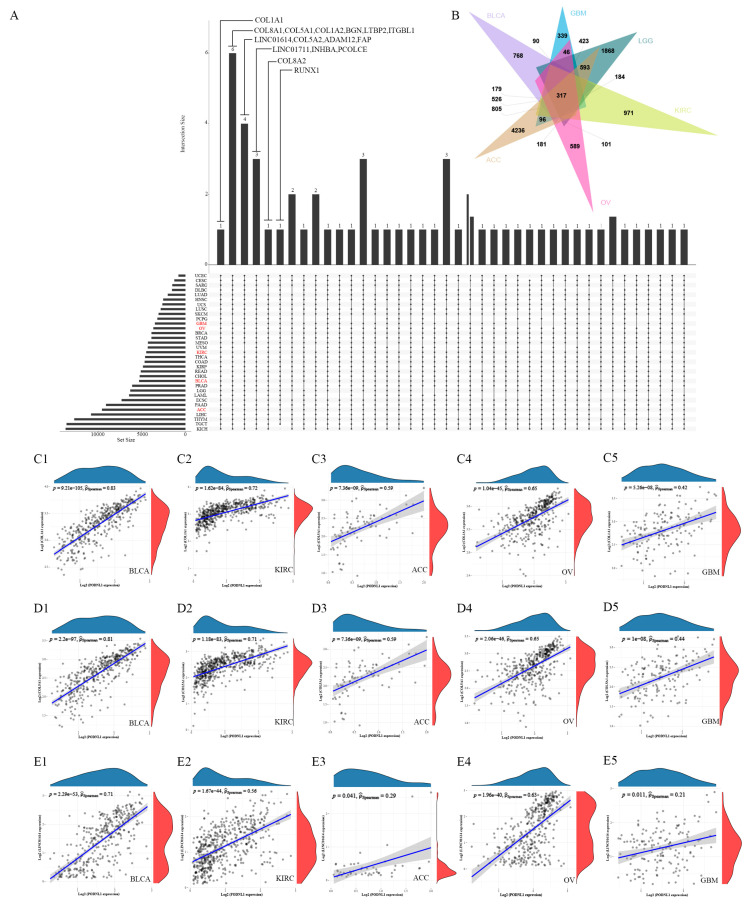
Expression correlation analysis of PODNL1 at the whole genome level in pan-cancer. (**A**) Upset plot showed the genes correlated with *PODNL1* expressions in pan-cancer. (**B**) Venn plots showed the genes correlated with *PODNL1* expressions in five types of cancers including BLCA, KIRC, ACC, OV and GBM. (**C1**–**E5**) The expression correlations of *PODNL1* with (**C1**–**C5**) *COL1A1*, (**D1**–**D5**) *COL5A1*, and (**E1**–**E5**) LINC01614 in BLCA, KIRC, ACC, OV and GBM, respectively.

The genome-wide co-expression analyses showed that the expression levels of collagen genes (COL1A1, COL1A2, COL5A1, COL5A2, COL8A1 and COL8A2) and LincRNAs (Linc01614 and Linc01711) were significantly positively correlated with PODNL1 expressions in various cancers (Figure 12). Meanwhile, in drug responses, we found that PODNL1 expressions were positively correlated with the clinical responses after treating with BEN, Tegafur, Lenvatinib, Megestrol acetate, and okadaic acid, but negatively in AT-13387 and Allopurinol treatments (Figure S1).

### 3.10. TMA-Based Multiplex Immunofluorescence Staining

**Figure 13:**
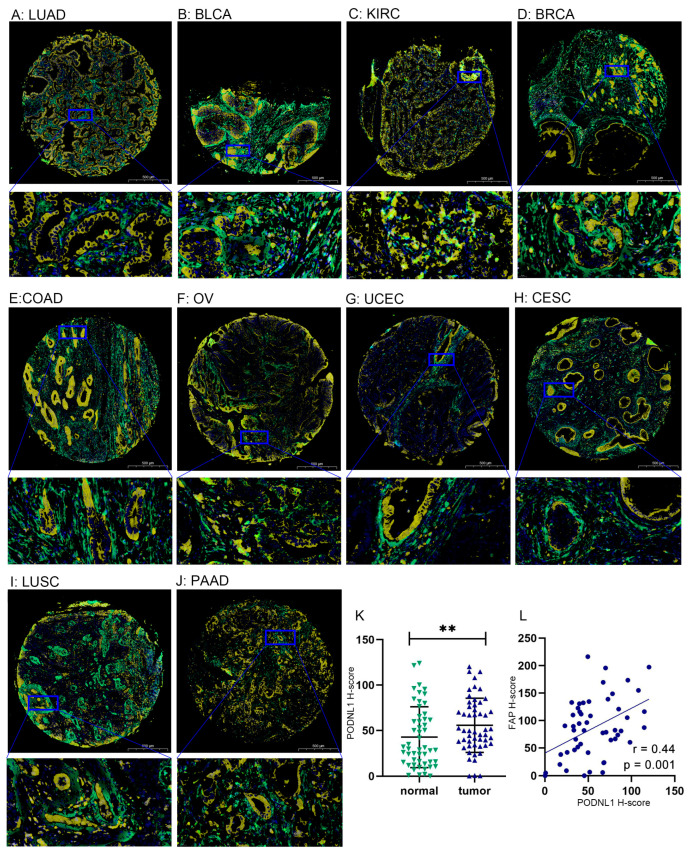
The digital image analyses of the relationship between the protein levels of PODNL1 and FAP using the TMA-based multiplex immunofluorescence staining detected from 52 paired samples of 20 types of cancer. (**A**–**J**) The merged images of the multiplex immunofluorescence staining (50× and 500×), the DAPI nuclear is stained in blue, while the protein levels of PODNL1 and FAP, respectively, are stained in yellow and green; (**A**) LUAD; (**B**) BLCA; (**C**) KIRC; (**D**) BRCA; (**E**) COAD; (**F**) OV; (**G**) UCEC; (**H**) CESC; (**I**) LUSC; (**J**) PAAD; (**K**) Comparison of the PONDL1 H-scores between the pairs of tumor and matched adjacent normal tissues; (**L**) The relationship between the PONDL1 and FAP H-scores. ** *p* < 0.01.

Using the HPA database for further analysis, we found that the majority of malignancies displayed cytoplasmic positivity of PODNL1 expression. Among them, the colorectal and thyroid cancers along with several cases of gastric, pancreatic and ovarian cancers exhibited stronger immunoreactivity (Figure S2). Furthermore, the DIA of TMA-based multiplex immunofluorescence staining revealed the protein co-expression relationship between PODNL1 and FAP, an important CAFs marker, in 20 types of cancer (Figure 13, Table S2). We found that both the protein levels of PODNL1 and FAP were higher in TME, and PODNL1 protein was especially highly expressed in cancer cells (Figure 13A–J). Meanwhile, it was found that the H-score of PODNL1 in tumor tissues was significantly higher than that in adjacent normal tissues (Figure 13K). The H-score of PODNL1 was significantly positively correlated with FAP (Figure 13L).

## 4. Discussion

PODNL1 is an important member of the SLRP family which includes ubiquitous ECM components involved in matrix organization and signaling regulation in TME [4]. At present, a number of studies have revealed the promoting role of PODNL1 in tumor proliferation and EMT [14,15,16,17,18]. In high-grade glioma cell lines, PODNL1 has been demonstrated to stimulate cell proliferation and migration via the regulation of the Akt-mTOR axis [16]. In the present study, we found that *PODNL1* was highly expressed in tumor tissues in 15 types of cancers and its high expression was significantly associated with more advanced stages and poor prognosis in multiple types of cancers. Notably, *PODNL1* expressions were also enriched in more aggressive molecular subtypes in various tumors, such as the mesenchymal type of GBM [29] and OV [30], and M3 type of KIRC [31]. Combined with previous studies [14,15,16,17,18], such results indicate that *PODNL1* could serve as a novel potential biomarker to guide prognosis predictions and individualized therapies in cancers.

The immune infiltration in TME involves interplay of various cell types and complex signaling pathways. The cell types like cancer cells, stromal cells, endothelial cells, and immune cells interplay in TME, driving cancer progression, prognosis, and immunotherapeutic responses [32]. Our analyses indicated that the *PODNL1* expression was notably correlated with immune infiltration in TME. Specifically, it was significantly positively correlated with the infiltration levels of CAFs in all types of cancers and other cells like endothelial cells, macrophages, MDSCs, and tumor-associated HSCs in most types of cancers. The CAFs play an important role in tumor promotion involving ECM remodeling and cancer stemness sustaining via the production of varieties of cytokines, growth factors, and cell-surface molecules [32,33]. In the other case, MDSCs, as pathologically activated immature cells, can suppress T-cell activity and contribute to immunosuppression in TME as well [34]. Our study further demonstrated that the high expression levels of PODNL1 were positively correlated with the molecular signatures and hallmarks of tumors including Pan-TBR, TGF-β, TNF-α, inflammatory response, apical junction, coagulation, EMT, and hedgehog. Current immune–checkpoint blockade (ICB)-based immunotherapies, commonly targeting PD-1, PD-L1 and CTLA-4, have not achieved the desired efficacy in the therapeutic practices in different types of cancers, suggesting that tumor cells may develop other ways to evade immune surveillance. Recent report indicates that targeting CD276 may enhance ICB-based immunotherapy in HNSC because it overexpressed in cancer stem cells and could be utilized to escape attacking from CD8^+^ T cells [35]. Interestingly, our study showed a concordant indication by presenting a positive correlation between *CD276* and *PODNL1* expressions in pan-cancer. Put together, all these results suggested that the high expression levels of PODNL1 correlated with various cells infiltration levels and molecular signaling in TME, may involve ECM remodeling, uncontrolled inflammation activation and maintenance of immune-suppressive signaling. Further analyses on the correlations with the clinical responses of ICB also indicated its potential predictive value on tumor immunotherapy.

*TGF*-β signaling plays a critical role in multiple biological processes including inflammation, immunosuppression, angiogenesis, metastasis, EMT, fibroblast activation and desmoplasia in TME. But, this signaling pathway can be both tumor-promoting and tumor-suppressing, which is called the “*TGF*-β paradox” [36,37]. Increasing evidence shows that the transferring of downstream *TGF*-β signaling from the canonical SMAD2/3:4 signaling cascade to non-canonical cascades (such as the mitogen-activated protein kinases (MAPKs), PI3K/AKT, rhodopsin (Rho) and TNF receptor-associated factor (TRAF) 4/6) may be a critical trigger for tumor promotion [38,39]. Importantly, our study found that *PODNL1* high expression was significantly correlated with downregulations of the SMAD2/3:4 heterotrimer regulations transcription and upregulations of the pathway-restricted SMAD protein phosphorylation. Meanwhile, GSVA of cancer hallmark gene sets showed positive correlation with the PI3K-AKT-mTOR pathway, indicating that *PODNL1* may involve regulating the switch of downstream pathways to induce pro-tumor effects. It is found that many members of the SLRP family are able to bind to and modulate receptors of bone morphogenetic protein (BMP)/TGF pathways [4]. Further PPI network analyses in this study indicated that PODNL1 protein shared similar domain with LRG1 of the LRR family, which has been verified to bind to and interact with the receptors of TGF-β signaling including ACVRL1, ENG and TGFBR1, and is involved in the progression of cancer and inflammatory disorders [40].

Moreover, *TGF*-β pathway activity in fibroblasts has been found significantly correlated with non-response in immunotherapies in the mUC anti-PD-L1 cohort. The *TGF*-β-activated CAFs are observed to be significantly associated with cancer immune evasion. A large-scale pan-cancer analysis indicates that the presence of a *TGF*-β-associated C-ECM signature including a distinct set of ECM genes upregulated in cancer links CAFs to immune evasion and immunotherapy failure [41]. In the groups with high expression levels of PODNL1, we observed the extensive enrichment of CAFs in all 33 types of cancers and significant inhibition of canonical cascades and activation of non-canonical cascades of *TGF*-β signaling. Then, our further analyses of single-cell transcriptomes showed that *PODNL1* expression was mainly enriched in the cancer cells of BLCA, GBM, HNSC, KIRC, and OV, and in the CAFs in BLCA and HNSC as well. Interestingly, through the TMA-based multiplex immunofluorescence staining, we further validated that the PODNL1 protein was especially highly expressed in cancer nests and positively correlated with FAP expression in TME in 20 types of cancer, which has been known as an important marker of activated CAFs and was verified as being associated with fibrosis and ECM degradation, and strongly expressed in the tumor stroma [42]. Previous studies showed that the knockdown of *PODNL1* in the cell lines of GBM and BLCA can significantly influence the malignant biological behavior of tumor cells, including EMT and high proliferation [17,18]. Similarly, herein, *PODNL1* was demonstrated to be involved in maintaining the CAF-TGF-β signaling loop in TME, and then might promote the continuous activation of EMT cascades and generated tumor immune evasion.

Collagens and proteoglycans are fundamental elements in ECM [3]. Particularly, the positive correlations of *PODNL1* expressions with such collagens as collagen type 1 alpha 1 chain (*COL1A1*), *COL1A2* and collagen type V alpha 1 chain (*COL5A1*) were found in over 30 types of cancers. Chen et al. illustrate that cancer cell derived dysfunctional COL1A1 homotrimers can lead remodeling of the tumor microbiome, diminishing T-cell infiltration, and reducing anti-PD-1 immunotherapeutic response [43]. The COL5A1 is also reported as functionally prominent in the collagen hierarchy and fibers assembly in ECM and promoting proliferation and metastasis in cancers [44]. In short, our results indicate that *PODNL1* may involve collagen dysfunction in ECM remodeling in TME in a pan-cancer setting. 

The ECM crosslinks the genotype–phenotype cross-talk during malignancy [3]. This is also testified with our findings in the present study that the heterogeneity of cancer genotype–phenotype cross-talking was associated with *PODNL1*, the important SLPR member in ECM. In ACC, BLCA, HNSC, and KICH, the groups of high PODNL1 expressions were found with more *TP53* mutations. The wild-type *p53* can interact with the tumor-suppressive SMADs genes, but the mutant *p53* can subvert the tumor-suppressing responses of TGF-β signaling [45]. In pancreatic cancer, the *p53* mutation promotes the alternating activation of CAFs and ECM remodeling in TME and facilitates malignant progression [46].

Epigenetic modifications may reshape the TME [47]. The *PODNL1* methylation status has been reported to impact the prognosis of patients with LGG. In our study, the methylation level of the *PODNL1* site CG10729062 was found to be associated with OS in various cancers. Furthermore, Linc01614 was also found significantly positively co-expressed with *PODNL1* in multiple types of cancers. Linc01614, speculated as a biomarker for poor prognosis in breast cancer, is significantly upregulated in various tumor tissues and highly correlated with *TGF*-β signaling and ECM remodeling [48]. Put together, both the methylation and the related LincRNAs may participate in the complex regulatory network of *PODNL1* during tumor progression.

## 5. Conclusions

The present study comprehensively uncovered the correlations of the class V SLRP member *PODNL1* expression with the prognosis, immunotherapeutic response, TME immune infiltration, co-expression networks, mutation landscape and epigenetic regulation in a pan-cancer setting. Our systematic study indicates that *PODNL1* promotes tumor progression by activating tumor-promoting *TGF*-β signaling, involving the formation and maintenance of uncontrolled inflammatory EMT and ECM remodeling in the TME. Our study has laid a foundation for further elucidating the molecular mechanisms of *PODNL1* related regulatory network in tumor progression and developing *PODNL1* as a potential tumor matrix-mediated biomarker for immunotherapy and prognosis in a pan-cancer setting.

## Data Availability

Data supporting the conclusions of this article are included within the article and its additional files.

## Supplementary Materials

The following supporting information can be downloaded at https://www.mdpi.com/article/10.3390/cimb45070386/s1: Table S1: List of abbreviations in this study; Table S2: Tissue microarray analyses of the relationship between protein levels of PODNL1 and FAP via multiplex immunofluorescence staining detected from 52 paired samples of 20 types of cancer; Figure S1: Correlation plots between *PODNL1* expression and IC50 of drugs analyzed using the Cellminer datasets. Figure S2: Immunohistochemistry images of tumor tissues showed moderate cytoplasmic positivity of PODNL1 (antibody HPA042807) analyzed through the HPA database.
