# Supplementary material for: Small Leucine-Rich Proteoglycan PODNL1 Identified as a Potential Tumor Matrix-Mediated Biomarker for Prognosis and Immunotherapy in a Pan-Cancer Setting
Source: Curr Issues Mol Biol. 2023 Jul 22;45(7):6116–39. doi: 10.3390/cimb45070386 (PMC10378008; doi:10.3390/cimb45070386)
Supplement: Supplementary file 1 [file cimb-45-00386-s001.zip › cimb-2492160-supplementary.pdf]

Small leucine-rich proteoglycan PODNL1 identified as a promising tumor matrix-mediated biomarker for prognosis and immunotherapy in a pan-cancer setting

Geyang Dai [1,2], Yue Sun [1,2], Rui Wei [1,2] and Ling Xi [1,2,*]
Correspondence: Ling Xi (lxi@tjh.tjmu.edu.cn)

Table S1. List of abbreviations

Abbreviation	Full name
ACC	Adrenocortical carcinoma
ACVRL1	Activin A Receptor Like Type 1
AKT	The serine/threonine protein kinase
APM	Antigen processing machinery
AUC	The area under the ROC curves
BLCA	Bladder urothelial carcinoma
BMP	Bone morphogenetic proteins
BRAF	V-raf murine sarcoma viral oncogene homolog B1
BRCA	Breast invasive carcinoma
CAFs	Cancer-associated fibroblasts
CCLE	Cancer Cell Line Encyclopedia
CCND1	Cyclin D1
CD276	Cluster of differentiation 276
CD44	Cluster of differentiation 44
CESC	Cervical squamous cell carcinoma and endocervical adenocarcinoma
CHOL	Cholangiocarcinoma
CIBERSORT	Cell-type Identification By Estimating Relative Subsets Of RNA Transcripts
COAD	Colon adenocarcinoma
COL1A1	Collagen type I alpha 1 chain
COL1A2	Collagen type I alpha 2 chain
COL5A1	Collagen Type V Alpha 1 Chain
CR	The complete response
CTL	the cytotoxic T lymphocytes
CTLA4	Cytotoxic T-Lymphocyte associated protein 4, also named as cluster of differentiation 152, CD152
CTNNB1	Catenin beta-1
DDR	DNA_damage_ response

DFS	Disease-free survival
DLBC	Lymphoid neoplasm diffuse large B-cell lymphoma
DSS	Disease-specific survival
ECM	Extracellular matrix
EMT	Epithelial-mesenchymal transition
ENG	Endoglin
EPIC	Estimating the Proportion of Immune and Cancer cells
ERK	Extracellular signal-regulated kinase
ESCA	Esophageal carcinoma
ESTIMATE	Estimation of Stromal and Immune Cells in Malignant Tumor Tissues Using Expression Data
GBM	Glioblastoma multiforme
GEO	The Gene Expression Omnibus
GEPIA2	The Gene Expression Profiling Interactive Analysis
GSEA	Gene Set Enrichment Analysis
GSVA	Gene set Variation analysis
GTEx	The Genotype-Tissue Expression
GTF2I	General Transcription Factor IIi
HNSC	Head and neck squamous cell carcinoma
HPA	The Human Protein Atlas
HSCs	Hematopoietic stem cells
ICB	Immune-checkpoint blockade
IHC	Immunohistochemistry
IL	Interleukin
JAK	Janus kinase
KEGG	Kyoto Encyclopedia of Genes and Genomes
KICH	Kidney chromophobe
KIRC	Kidney renal clear cell carcinoma
KIRP	Kidney renal papillary cell carcinoma
K-M	Kaplan-Meier
LAML	Acute myeloid leukemia
LGG	Brain lower grade glioma
LIHC	Liver hepatocellular carcinoma
LincRNAs	The long coding RNAs
LRG1	Leucine-richα-2 glycoprotein 1
LRP-1	The low-density lipoprotein receptor-related protein
LRR	Leucine rich repeats
LUAD	Lung adenocarcinoma
LUSC	Lung squamous cell carcinoma
MAPKs	The mitogen-activated protein kinases
MCODE	The Molecular Complex Detection

MCP-counter	The Microenvironment Cell Populations-counter
MDSCs	Myeloid-derived suppressor cells
MESO	Mesothelioma
MET	Hepatocyte growth factor receptor
MHC	Major histocompatibility complex
MSI	Microsatellite Instability
MsigDB	Molecular signatures database
mTOR	Mechanistic Target Of Rapamycin Kinase
mUC	Metastatic urothelial cancer
NACC1	Nucleus accumbens-associated protein 1
NES	Nestin
NK cells	Natural killer cells
NOTCH1	Neurogenic locus notch homolog protein 1
NOTCH4	Neurogenic locus notch homolog protein 4
OS	Overall survival time in day
OV	Ovarian serous cystadenocarcinoma
PAAD	Pancreatic adenocarcinoma
Pan_F_TBRs	Pan-fibroblast TGF-β response signature scores
PCA	Principal component analysis
PCPG	Pheochromocytoma and paraganglioma
PD	Progressive disease
PD-1	Programmed cell death protein-1
PD-L1	Programmed cell death-ligand 1, also known as cluster of differentiation 274, CD274
PFS	Progression-free survival
PI3K	Phosphoinositide 3-kinases
PIK3CA	Phosphatidylinositol-4,5-bisphosphate 3-kinase catalytic subunit alpha
PODNL1	Podocan Like 1
PPI	The protein-protein interaction
PR	Partial response
PRAD	Prostate adenocarcinoma
quanTIseq	A method to quantify the fractions of ten immune cell types from bulk RNA-sequencing data
RAF	Proto-oncogene serine/threonine-protein kinase
READ	Rectum adenocarcinoma
REXO1	RNA exonuclease 1 homolog
Rho	Rhodopsin
ROC	The Receiver Operating Characteristic
SALL1	Spalt Like Transcription Factor 1
SARC	Sarcoma
SD	Stable disease

SKCM	Skin cutaneous melanoma
SLRPs	Small leucine-rich proteoglycans
SMADs	The small mother against decapentaplegic proteins (SMADs)
SOX2	Sex determining region Y-Box Transcription Factor 2
STAD	Stomach adenocarcinoma
STAT	The signal transducer of activators of transcription
TCGA	The Cancer Genome Atlas
TGCT	Testicular germ cell tumors
TGFBR1	Transforming Growth Factor Beta Receptor 1
TGF-β	Transforming growth factor beta
THCA	Thyroid carcinoma
THYM	Thymoma
TIDE	Tumor Immune Dysfunction and Exclusion
TIMER	Tumor Immune Estimation Resource
TISDB	An integrated repository portal for tumor-immune system interactions
TMB	Tumor mutation burden
TME	Tumor microenvironment
TNF-α	Tumor necrosis factor -α
TRAF	The tumor necrosis factor receptor-associated factor
Tregs	Regulatory T-cells
TRK	The tropomyosin receptor kinase
UCEC	Uterine corpus endometrial carcinoma
UCS	Uterine carcinosarcoma
UMAP	Uniform Manifold Approximation and Projection
UVM	Uveal melanoma

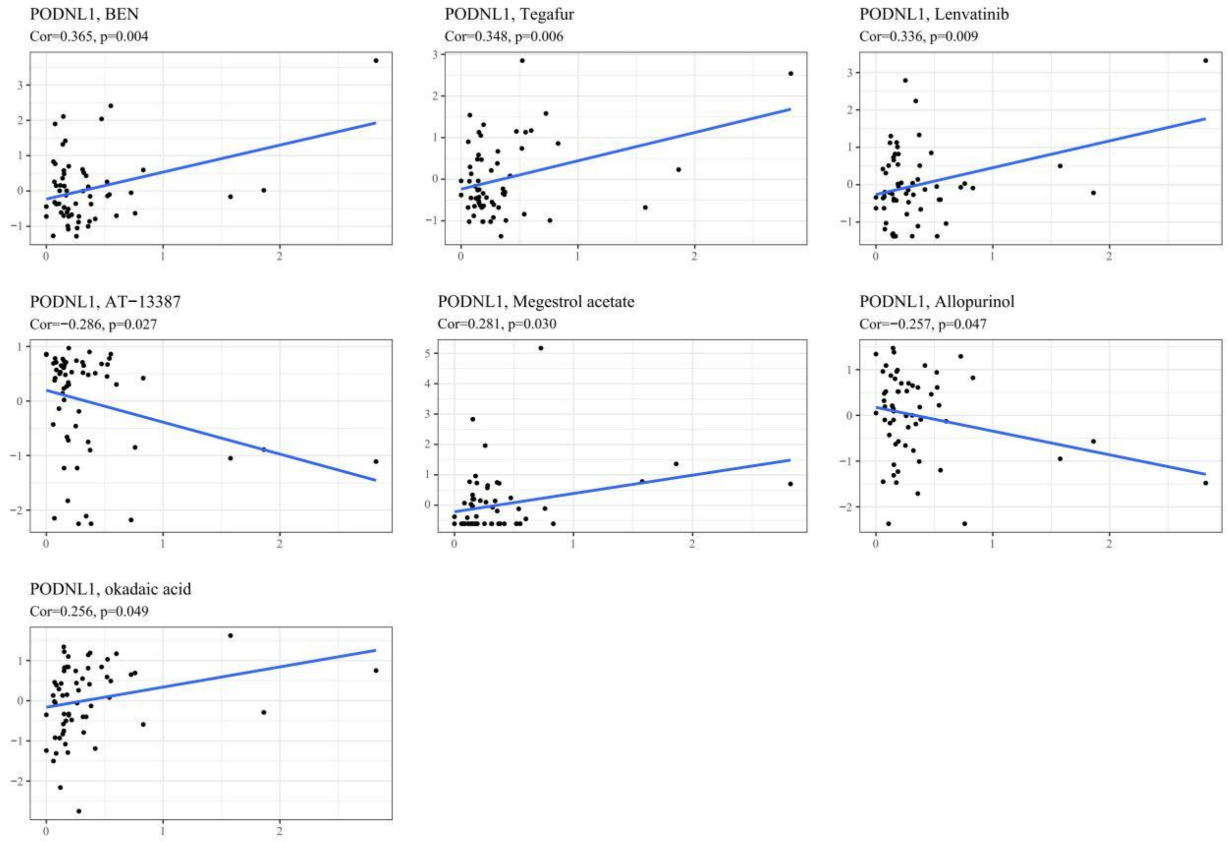

Figure S1. Correlation plots between PODNL1 expression and IC50 of drugs, including BEN, Tegafur, Lenvatinib, AT-13387, Megestrol acetate, Allopurinol, Okadaic acid.

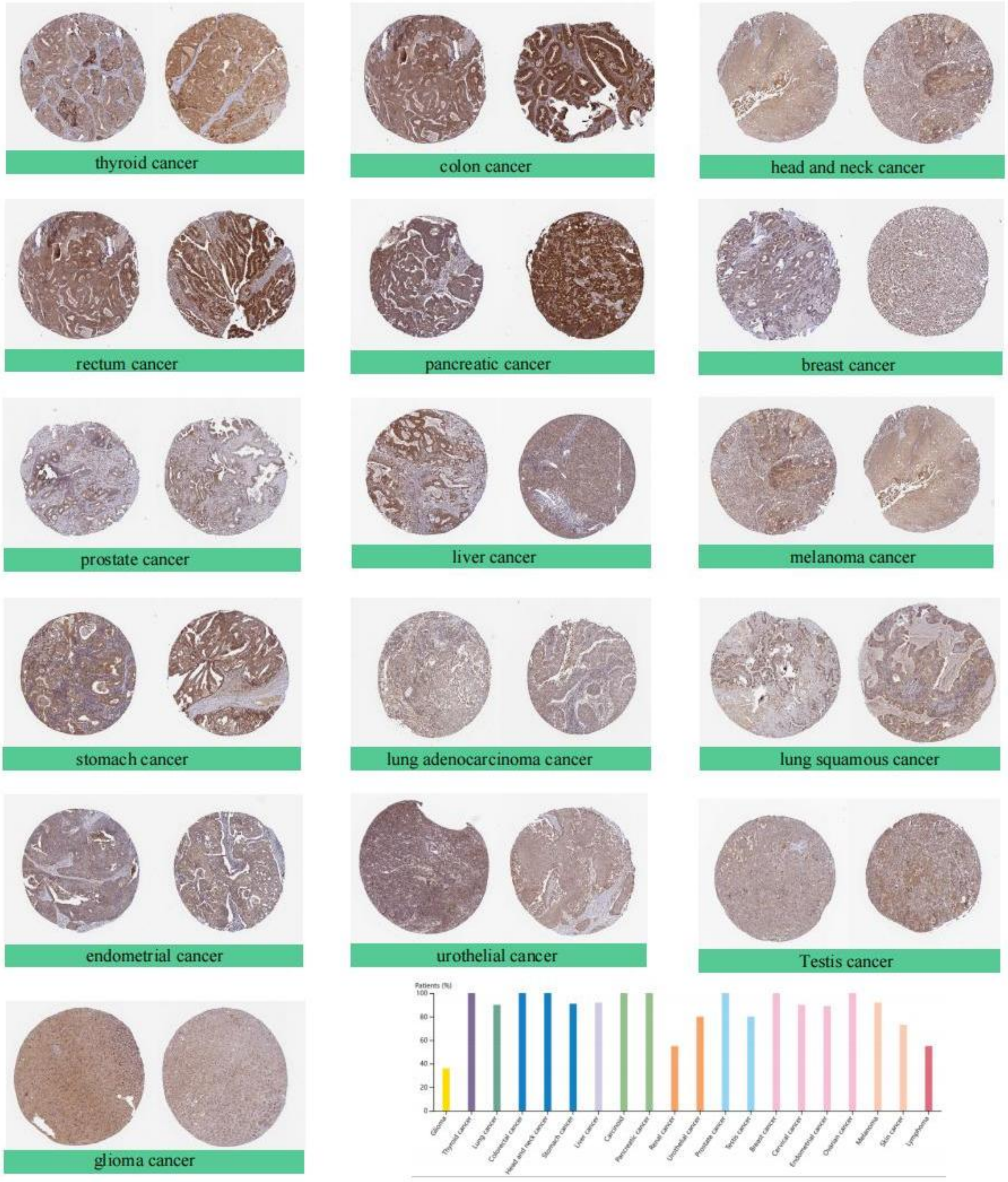

Figure S2. Immunohistochemistry images of tumor tissues showed moderate cytoplasmic positivity of PODNL1 (antibody HPA042807), including (**A**) thyroid cancer, (**B**)colon cancer, (**C**)head and neck cancer, (**D**)rectum cancer, (**F**)pancreatic cancer, (**G**)breast cancer, (**H**)prostate cancer, (**I**)liver cancer, (**J**)melanoma cancer, (**K**)stomach cancer, (**L**)lung adenocarcinoma, (**M**)lung squamous cancer, (**N**)endometrial cancer, (**O**)urothelial cancer, (**P**)testis cancer and (**Q**)glioma.
